# Beyond the skin surface: untangling sensitive skin syndrome and peripheral neuropathy through an exploratory real-world data analysis

**DOI:** 10.3389/fpain.2026.1883218

**Published:** 2026-10-12

**Authors:** Miranda A. Farage, Inna Eiberger

**Affiliations:** 1 The Procter and Gamble Company, Mason, OH, United States; 2 DermatoSciences, Cincinnati, OH, United States; 3 The Procter and Gamble Company, Schwalbach am Taunus, Germany

**Keywords:** altered skin sensation, cardiovascular disease, comorbidities, diabetes, misdiagnosis, sensitive skin, peripheral neuropathy, real-world data

## Abstract

**Introduction:**

Sensitive skin syndrome (SSS) is commonly reported despite its nonspecific definition, whereas peripheral neuropathy (PN) remains underrecognized in the general population. Because localized paresthesia and numbness—particularly in the hands and feet—may be perceived as skin problems, some patients reporting sensitive skin could have unrecognized neuropathy. This exploratory real-world evidence study investigated the potential overlap between SSS and PN.

**Methods:**

Patient-level real-world data from health records of individuals reporting sensitive skin were analyzed to characterize associated diagnoses, symptoms, comorbidities, and risk factors potentially related to PN.

**Results:**

Diagnoses among patients reporting sensitive skin included dermatitis (44%), altered skin sensation (33%), dry skin or urticaria (15%), and acne (13%). Within the group diagnosed with altered skin sensation, 18% reported paresthesia or tingling, 11% reported anesthesia or numbness, and 4% reported other symptoms. Documented comorbidities and risk factors included established contributors to PN, such as diabetes, cardiovascular disease, and smoking.

**Discussion:**

The findings suggest a potential diagnostic overlap between SSS and PN and raise the hypothesis that some patients with altered skin sensation may have unrecognized PN. They also demonstrate how real-world data can reveal symptom patterns that may be overlooked during routine clinical assessment. Because the hypothesis arose from the same dataset used to explore it, these findings should be considered hypothesis-generating and require prospective confirmation. Nevertheless, timely differentiation between SSS and PN could facilitate appropriate clinical evaluation and improve patient health and well-being.

## 1 Introduction

While sensitive skin is commonly reported by patients, though not clearly defined, peripheral neuropathy (PN) is not well known to the general population, and the two conditions seem not to be connected at first glance. The current real-world evidence (RWE) study provides new insights into the potential relation between sensitive skin syndrome and PN.

Peripheral neuropathy (PN) is the most common disorder of the peripheral nervous system (PNS) in adults and may affect up to 15% of the general population aged over 40 years across the globe (1–3). PN can be caused by damage to the peripheral nerves as a result of various underlying conditions and/or risk factors, including diabetes, obesity, hereditary disorders, chemotherapy, Vitamin B deficiencies, infections, cancer, or alcohol (4–7). With a share of up to 53%, diabetes is unambiguously the most common cause of PN in the world, followed by idiopathic PN in approximately one quarter of cases where the reason remains unclear (4, 8, 9). Typical sensory symptoms of PN are numbness, tingling, pins-and-needles or electric shock-like sensation, and burning pain, mostly in the limbs, starting in the feet and progressing to hands (5, 7, 10). If PN is not treated, the disease usually progresses and becomes a major burden for patients at some point due to physical, emotional, and social restrictions (11–14). Furthermore, PN is associated with the risk of serious complications such as foot ulcers (15–17), which also negatively impact quality of life and, as severity increases, place an increasing burden on healthcare systems (18, 19). Early detection and treatment of PN are essential to avoid the progression of PN and associated individual and societal burdens (16, 20). Despite its high prevalence in the general population, PN is still often deprioritized in favor of other disease complications and remains unattended and undiagnosed, resulting in approximately 80% of patients with PN who never receive a formal diagnosis (3, 21, 22). The reasons for this high number are various for both patients and healthcare professionals (HCPs). In patients, this is mainly due to a lack of awareness of the disease and its signs, difficulties in describing their symptoms, as well as the fact that PN usually develops insidiously and the symptoms are initially barely noticed or attributed to irrevocable circumstances such as aging, which is why patients hesitate to report symptoms (21, 23). As symptoms are localized, patients also tend to misinterpret them for localized joint or skin conditions, for example. Physicians often have limited time per patient, lack diagnostic routine or are unaware of simple screening and diagnosis tools, which is partly driven by the lack of harmonized guidelines for PN diagnosis (16, 21, 23). Objective measures to diagnose PN, such as corneal confocal microscopy (CCM) and skin biopsy, exist, although these are not available to the majority of physicians and are currently mainly used in clinical research (24–26). Additionally, when dealing with multiple conditions, physicians may underestimate the serious consequences of PN for the general health and well-being of their patients and prioritize, for example, the management of hyperglycemia in their diabetic patients, while they assume that the benefit of early recognition and treatment of PN is limited anyway (16, 21, 23). Accordingly, numerous attempts are currently being made to promote the earlier diagnosis of PN in primary care, even in resource-poor settings, and thus prevent the progression of unrecognized PN to an advanced stage, where patients first present when foot amputation is already unavoidable (21, 23).

Sensitive skin syndrome (SSS) is characterized by uncomfortable skin sensations such as itching, burning, stinging, tingling, or pain in response to usually non-irritating stimuli (27–29). According to numerous surveys, up to ∼70% of the general population shows some degree of sensitive skin, with women being more frequently affected than men (29–31). Accordingly, female gender is clearly considered an intrinsic risk factor for sensitive skin, whereas there are somewhat conflicting results regarding the role of age (32). While the prevalence of self-reported sensitive skin is higher in younger than in older individuals, older subjects report an increase in the severity of their skin sensitivity (32, 33). However, the decrease in prevalence with increasing age applies primarily to men, with some surveys indicating opposite effects among women (33). At the same time, the prevalence of sensitive skin seems to differ between anatomical sites (i.e., face, body, or genital area) (32, 33). These findings indicate that no individual risk factors exist; rather, several factors need to be considered in interaction. Ethnicity, comorbidities, hormonal status, and others are discussed as further intrinsic factors affecting the risk of sensitive skin (27). Extrinsic risk factors for skin sensitivity include, among others, exposure to consumer products, weather conditions, air conditioning, and cultural influences (28, 29, 33).

As international consensus on diagnostic criteria or tests is lacking, for a significant amount of time SSS has been considered a medical concept or a cosmetic or even psychosomatic issue rather than a recognized dermatological condition (27, 29, 34). The diagnosis of SSS is currently based on subjective symptoms that are assessed using patient-reported scales, although some sensory tests can be used as supportive tools (35–37). Regarding its etiology, a current research approach assumes that, in some cases, SSS could be a form of ‘low-noise’ small-fiber neuropathy (SFN) associated with hypersensitivity of sensory nerve endings (and keratinocytes) to environmental factors (36, 38–40). However, final evidence is still pending. It is important to distinguish two phenotypes that are sometimes conflated: a gain-of-function pattern (hypersensitivity, tingling, and paresthesia) that characterizes much of the sensitive skin and small-fiber neuropathy (SFN) literature, and a loss-of-function pattern (numbness and anesthesia) that characterizes classic peripheral neuropathy. We do not propose a single mechanism; rather, we consider that patients reporting altered skin sensation may fall along this gain-to-loss spectrum. Notably, if SSS is itself a neuropathic condition, the boundary between SSS and PN may be better understood as a spectrum than a dichotomy. The present study cannot establish the direction of any association between the two, and this ambiguity is considered explicitly when interpreting our findings.

PN and SSS seem not to be related to each other at first glance and are often handled by different physicians or in separate consultations, even though patients perceive the similar symptoms of both conditions, such as burning, tingling, or stinging pain, and may misinterpret them. Altered skin sensation has a broad differential diagnosis beyond generalized PN, including localized neuropathic conditions such as notalgia paresthetica and brachioradial pruritus, entrapment neuropathies, radiculopathy, and medication or treatment effects. Several of these are themselves neurological in origin, which is consistent with a nerve-related interpretation, but they are distinct from length-dependent peripheral neuropathy and should be considered before symptoms are attributed to PN. Indeed, it is confusing for patients to differentiate the symptoms, know potential diseases or causes, and make an appointment with the right HCP. Both patient groups often search for solutions for a long time without receiving a diagnosis or treatment. If patients with PN mistakenly attribute their symptoms to a skin condition while the actual underlying condition remains untreated, nerve damage progresses, significantly affecting health and quality of life. Creating awareness of PN risk factors and symptoms, as well as implementing simple screening and diagnosis tools as a standard of care will help HCPs and patients to diagnose PN early and start adequate treatment (21, 23).

Based on the reviews of our patient-related real-world data (RWD) of sensitive skin, new findings emerged, leading us to hypothesize that there may be an association between SSS and PN. To address this question, we examined the RWD regarding characteristics such as demographics, diagnoses, and symptoms to generate RWE. RWE studies offer a cost-effective alternative to controlled trials and—when data are adequately analyzed—can provide reliable insights and increase the efficiency of medical research and decision-making (41). By using RWE, we also aim to identify patterns to facilitate diagnosis and improve patient care. The practical relevance of this work is to raise clinicians’ index of suspicion for peripheral neuropathy in patients who present with sensitive-skin complaints, particularly extremity-predominant symptoms in older patients, so that a treatable and progressive condition is not overlooked at the point of first encounter.

## 2 Methods

### 2.1 Study design and data collection

This retrospective study collected real-world data (RWD) from a variety of sources such as EPIC, CERNER, MediTech, MacroHelix, ScriptPro, AllScript, QS/1, etc. using Loopback Analytics. Data collection was performed between October 1, 2019, and September 30, 2022, by THREAD, Cary, NC, USA, while the analysis took place between March and June 2023. This collection window overlapped with the COVID-19 pandemic, which might have influenced health-seeking behavior, access to specialties, and coding practices during the study period (see Limitations). Integrated RWD datasets consisted of electronic health records (EHRs), claims databases, hospital data, and pharmacy data from the United States. Medical notes of a selected subpopulation were curated to obtain information on clinical characteristics, health-seeking behavior, treatments, and outcomes to generate RWE.

Sensitive skin’ was identified by a free-text search of the source records for the term ’sensitive skin’ combined with the ICD-10 diagnosis codes listed below. Searching source databases covering more than 160 million individuals returned 3,645,000 records, of which 59,702 entries were linked to the term ’sensitive skin’. A ‘complete’ dataset was defined as one with non-missing values for age, gender, ethnicity, state of residence, EHR notes, and claims. After this filtering, 2,333 subjects had complete datasets and were included in the final analysis (study flow diagram, Supplementary Figure S1). Because only complete records were retained, potential selection bias is acknowledged in the Limitations. Data were included if the patients had at least one of the following ICD-10 diagnoses according to the International Classification of Diseases 10th revision (ICD-10): ‘L20 atopic dermatitis’, ‘L23 allergic contact dermatitis’, ‘L24 irritant contact dermatitis’, ‘L25 unspecified contact dermatitis’, ‘L50 urticaria’, ‘R20 disturbances of skin sensation’ (previous ICD-9 code 782.0), ‘R21 rash’, ‘L29.x pruritus, unspecified’, ‘L50.9 urticaria, unspecified’, ‘L53.9 erythematous condition, unspecified’, ‘L85.3 xerosis cutis’, ‘L51 erythema multiforme’ and ‘L70 acne’. The index date was defined as the date when the patients received their first ICD-10 diagnosis code from their healthcare professional (HCP) +/- 14 days. The follow-up period was defined as the time up to 6 months after the index date.

To generate more in-depth data, a purposively selected, quota-sampled subset of 240 patients was selected for data curation of medical charts: 100 based on age criteria (50 from the age group 18–34 years, 25 from the age group 35–64 years, and 25 from the age group 65 + years); 100 based on ethnicity (25 each from the groups ‘Hispanic’, ‘non-Hispanic White’, ‘non-Hispanic Black/African American’, and ‘non-Hispanic other’); and 40 based on treatment criteria (20 each from the groups ‘treated’ and ‘untreated’). Because this subset was quota-sampled by age, ethnicity, and treatment status, proportions derived from it are descriptive and are not generalizable to the full cohort of 2,333.

Data collection adhered to all regulations and ethical guidelines to ensure patient privacy and data security. This study analyzed a de-identified, retrospective dataset of medical records, from which all personal identifiers and non-clinical location markers had been removed prior to access. Because the analysis used only de-identified secondary data, it did not constitute human-subjects research and did not require Institutional Review Board review or informed consent.

### 2.2 Statistical analysis

Descriptive statistics summarized demographics, comorbidities, diagnostic categories and subcategories, symptoms, localization, and treatment. Datasets were grouped by variables such as age, gender, or ethnicity, and proportions were calculated. All analyses in this study are descriptive; no inferential statistical testing was performed, and therefore no group difference reported here should be interpreted as statistically significant. In particular, the study design does not permit a formal test of the hypothesized association between sensitive skin syndrome and peripheral neuropathy, and comparative statements are intended as descriptions of the observed proportions only. This limitation is stated explicitly in the Discussion.

## 3 Results

### 3.1 Demographic information and medical history

Of the 2,333 subjects with complete datasets, 68% (*N* = 1,596) were female and 32% (*N* = 737) were male. The age groups of SSS were 18–34 years (15%; *N* = 356), 35–54 years (35%; *N* = 823), 55–64 (22%; *N* = 503), and 65 + years (28%; *N* = 650). One person fell into the age group of 0–17 years. Most patients’ ethnicity was Caucasian (81%; *N* = 1900), followed by Black or African American (16%; *N* = 377). Most of the population resided in Southern states (47%; *N* = 1,101), followed by Western states (25%; *N* = 592) (Table 1). At the index date, almost half of the total population (48%) had no comorbidities or reported risk factors. In subjects with comorbidities and risk factors, cardiovascular disease (CVD; 26%) and type 2 diabetes mellitus (T2DM; 12%) were the two most common. Six months after the index date, only 16% of subjects had no comorbidity, while all comorbidities had become more common. CVD was still the most frequent comorbidity (50%), while the second most common was sleep disturbance (29%), followed by T2DM (22%) (Figure 1).

**Table 1:** Demographics.

	Total subjects 2,333	
Gender	%	N
Female	68	1,596
Male	32	737

Age group	%	N
0–17 years	0	1
18–34 years	15	356
35–54 years	35	823
55–64 years	22	503
65 + years	28	650

Race	%	N
White	81	1900
Black or African American	16	377
Asian	2	41
Native Hawaiian or other Pacific Islander	0.1	2
American Indian or Alaska Native	1	13

Residency in United States region*	%	N
South	47	1101
West	25	592
Mid-West	16	374
North-East	11	266

Data from electronic health records of *N* = 2,333 subjects. *The following states (two-letter abbreviations listed) were assigned to the following regions: West: WA, OR, CA, ID, NV, MT, WY, CO, NM, AK; Mid-West: ND, SD, NE, KS, MN, IA, MO, WI, IL, MI, IN, OH; South: OK, TX, AR, LA, TN, MS, AL, GA; NC, SC, FL, WV, VA, MD, DE; North-East: NY, PA, VT, MA, ME, NH, RI, CT, NJ. NA, not available.

**Figure 1:**
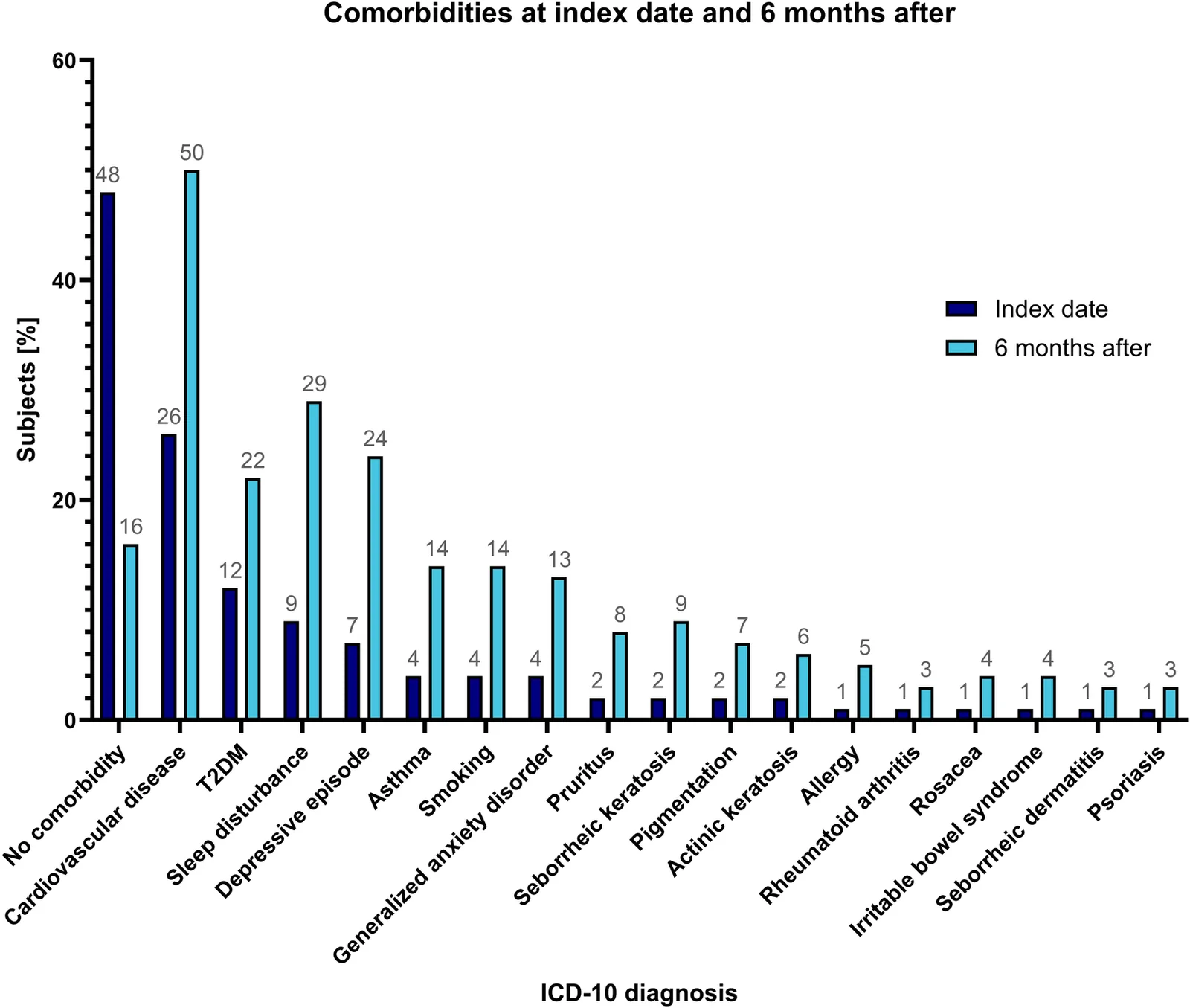
Comorbidities at index date* and 6 months after index date. Data from electronic health records of *N* = 2,333 subjects. *Index date was defined as the date when the patient received his/her skin-related ICD-10 diagnosis +/-14 days. ICD-10, International Classification of Diseases 10th revision

The increase in nearly every comorbidity between the index date and 6 months later could reflect either the accumulation of additional diagnostic codes over successive encounters or genuine new-onset disease. Because the dataset is de-identified and does not permit longitudinal tracking of same individual patients, these two explanations cannot be distinguished here, and the increase should be interpreted with that caveat (see Limitations).

### 3.2 Diagnostic categorization

Subjects received the first diagnosis at the index date when visiting the HCP. Overall, 44% of the total 2,333 subjects were diagnosed with dermatitis (29% unspecified dermatitis; 8% contact dermatitis, 7% atopic dermatitis), 33% with altered skin sensation, 15% with dry skin or urticaria, and 13% with acne (Figure 2A). For the consideration of SSS as a potential misinterpretation or subtype of PN, the diagnostic category of altered skin sensation was particularly relevant. In the category ‘altered skin sensation’, 18% across all age groups suffered from paresthesia, 11% from anesthesia, and 4% had other symptoms (Figure 2A). Analyzing the data by age revealed that the frequency of all subcategories rose with increasing age. In the oldest age group considered here (65 + years; dark blue bars), the prevalence of paresthesia was 21%, of anesthesia 13%, and of other signs 5%. In contrast, only 9% of the youngest age group (18–34 years; light blue bars) were affected by paresthesia, 7% by anesthesia, and 1% by other signs (Figure 2B).

**Figure 2:**
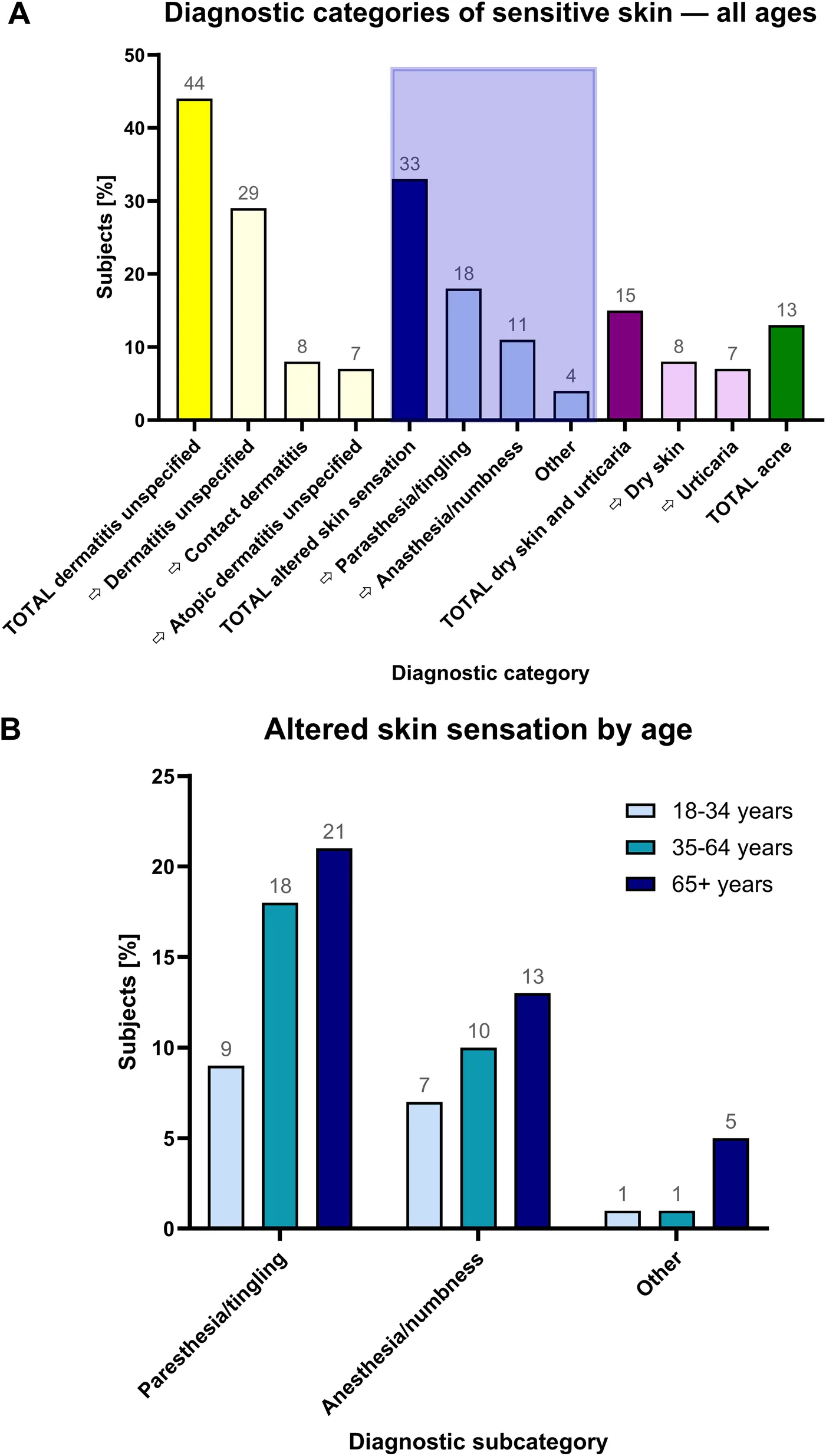
Diagnostic categories and subcategories for all ages and altered skin sensation by age. Data from electronic health records of *N* = 2,333 subjects. **A.** Diagnostic categories of sensitive skin—all ages. All diagnostic categories and subcategories are shown. **B.** Altered skin sensation by age. Only subcategories of ‘altered skin sensation’ are shown.

### 3.3 Specialties of healthcare professionals consulted for sensitive skin

About 47% of patients specifically visited the HCP for their SSS. Breaking down the specialties, 44% consulted family or general practice physicians, 9% internal medicine physicians, and 7% dermatologists. Other HCPs consulted included physician assistants (7%), nurses (7%), medical students (5%), and sports medicine physicians (2%). In 25% of cases the specialty was not identified in the record; because this section is used as supporting evidence, this substantial proportion of unknown specialties is an important caveat (Figure 3).

**Figure 3:**
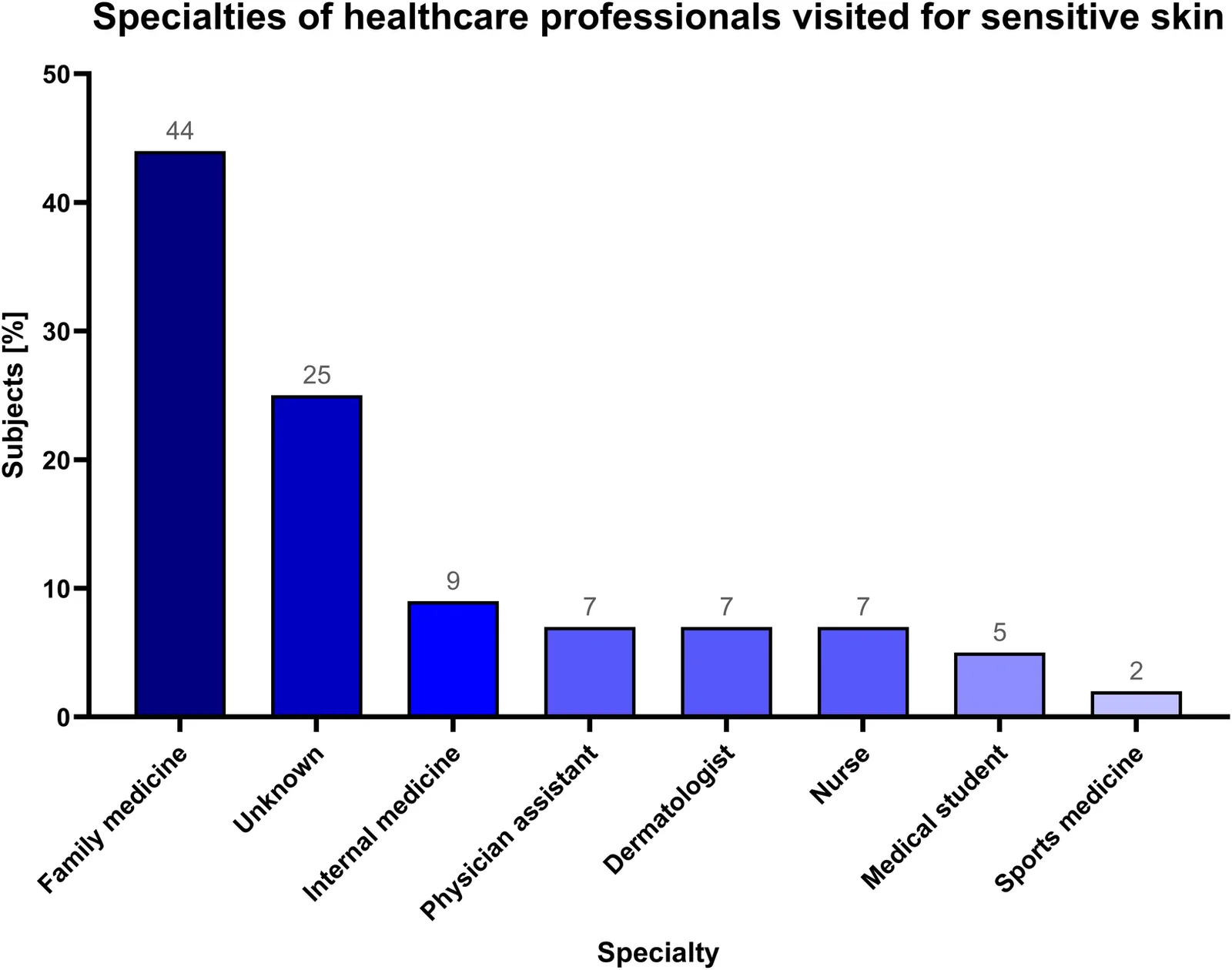
Specialties of healthcare professionals consulted for sensitive skin. Data from electronic health records of *N* = 2,333 subjects.

### 3.4 Symptoms

While the previous results derive from the total dataset of 2,333 subjects, the quota-sampled subset of 240 subjects selected for medical curation.

In the overall population (both genders together), altered skin sensation was reported more frequently in males (31%) than females (11%), while papules or macules were reported more frequently in females (18%) than males (5%); as these figures derive from the quota-sampled subset, they are presented descriptively (Figure 4A).

**Figure 4:**
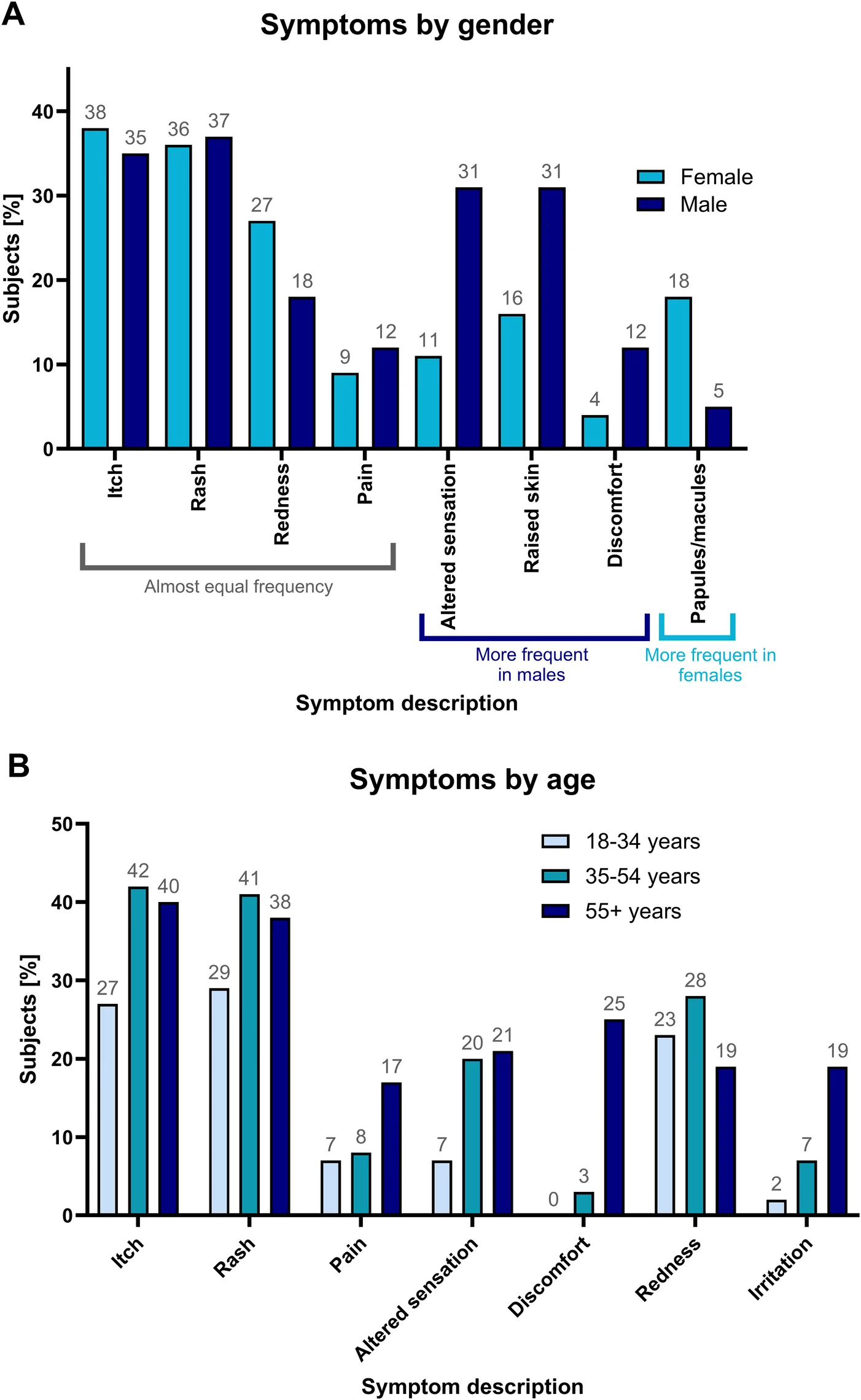
Symptoms by gender and age. **A.** Symptoms by gender. Data from the curation of the total *N* = 240 subjects. Symptoms were defined as more frequent in one gender when the prevalence was about twice as high or higher. **B.** Symptoms by age. Data from the curation of the age-stratified curated subset (*N* = 100), shown in the age bands 18–34 (*N* = 50), 35–54 (*N* = 25), and 55+ (*N* = 25) years.

Thermal sensory descriptors (loss of heat or cold sensation) and temperature-related complaints were not reliably or systematically recorded in the source data, and no quantitative thermal testing was available. Because thermal sensory loss is among the earliest features of small-fiber neuropathy, its absence from the record is itself informative and limits the confidence with which tingling and numbness alone can be attributed to PN (see Discussion and Limitations).

The prevalence of most symptoms increased with age and was lowest in the youngest age group, except for redness. This increasing trend was particularly evident for pain (18–34 years: 7%; 35–54 years: 8%; 55 + years: 17%), altered skin sensation (18–34 years: 7%; 35–54 years: 20%; 55 + years: 21%), discomfort (18–34 years: 0%; 35–54 years: 3%; 55 + years: 25%), and irritation (18–34 years: 2%; 35–54 years: 7%; 55 + years: 19%), for which the prevalence was numerically higher from the lowest age group to the second oldest or oldest age group (Figure 4B).

The prevalence of the individual symptoms also varied between the different ethnic groups. While itch was most frequent in White/Caucasian persons (60%), altered skin sensation and pain were particularly prevalent in Hispanics (32% and 20%, respectively), and raised skin was most common in Asians (20%). The only symptom with an almost equal distribution (range: 32%–40%) in all considered ethnicities was rash. Black/African Americans did not rank first for any of the symptoms (Supplementary Table S3).

Symptom severity was documented for only 11% of patients. Among these, 39% were mild, 39% moderate, and 21% severe; given the 89% missingness, this distribution should be interpreted with great caution and is not emphasized further.

### 3.5 Affected body areas

The localization of symptoms was also analyzed by age, gender, and ethnicity. As shown in Figure 5A, the face was the most affected body area in subjects aged 18–34 years (69%), whereas the frequency of facial symptoms declined in the two older groups (35–64 years: 22%; 65 + years: 30%). A marked increase in the two older age groups compared with the youngest age group was observed, among others, for arms (18–34 years: 13%; 35–64 years: 27%; 65 + years: 25%), legs (18–34 years: 11%; 35–64 years: 21%; 65 + years: 21%), hands (18–34 years: 6%; 35–64 years: 18%; 65 + years: 12%), and feet (18–34 years: 2%; 35–64 years: 8%; 65 + years: 10%).

**Figure 5:**
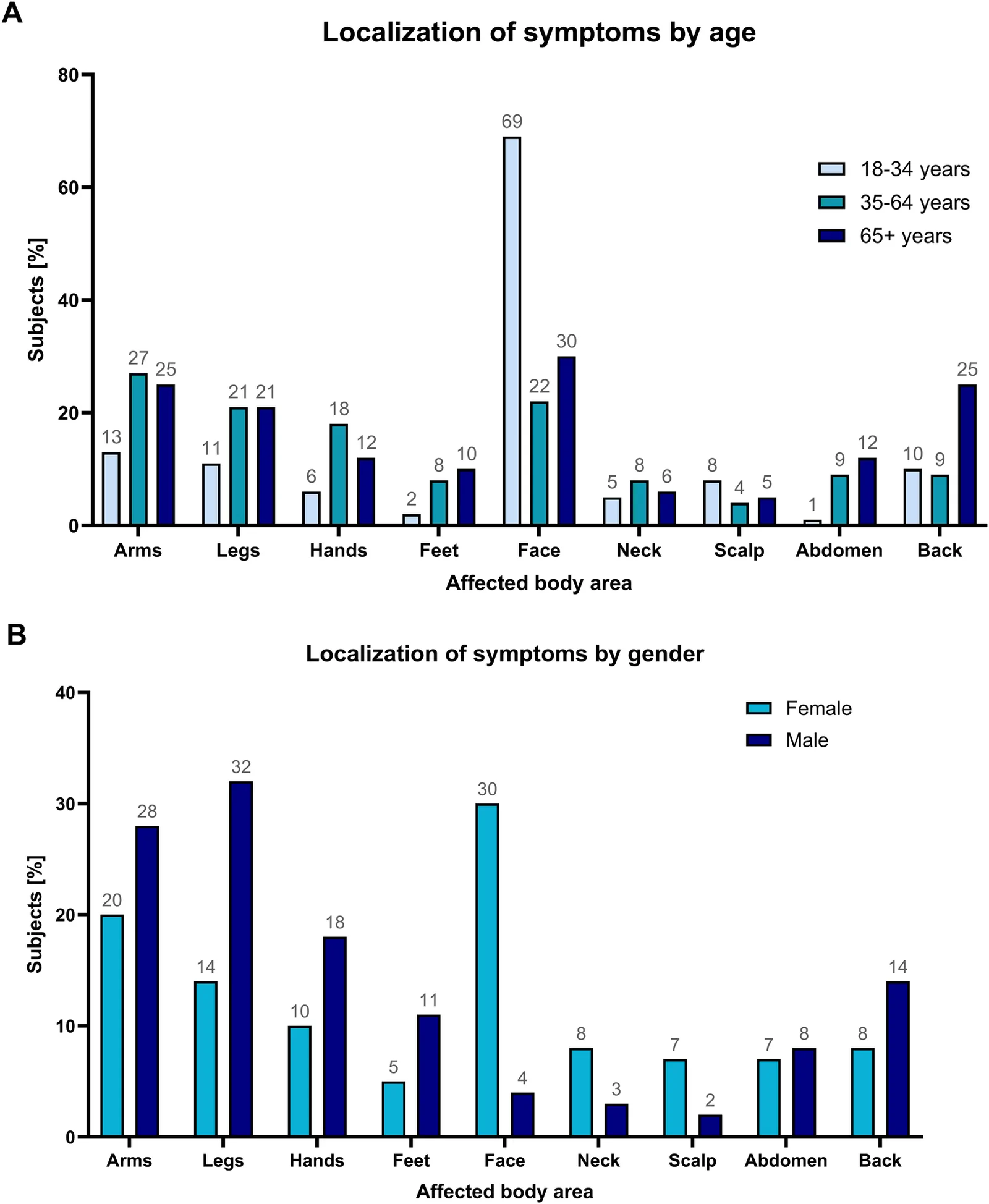
Localization of symptoms by age and gender. **A.** Localization of symptoms by age. Data from the curation of *N* = 50 subjects aged 18–34, *N* = 25 aged 35–64, and *N* = 25 aged 65 +. **B.** Localization of symptoms by gender. Data from the curation of the total *N* = 240 subjects.

There were also gender differences in the localization of symptoms. Overall, women were more frequently affected on the face than men (females: 30%; males: 4%), while men suffered from SSS symptoms more often on their arms (females: 20%; males: 28%), legs (females: 14%; males: 32%), hands (females: 10%; males: 18%), and feet (females: 5%; males: 11%) (Figure 5B).

Beyond that, ethnicity also influenced affected body regions. Overall, the face, arms, and legs were the three most affected body areas in all ethnic groups. Asians showed the highest prevalence of facial symptoms (44%), and symptoms on the legs (36%) were slightly more common than on the arms (32%). In Hispanics, the impact on body areas was more evenly distributed than in Asians, with the face (32%) leading and arms (24%) and legs (20%) being almost similarly affected.

Caucasians more often suffered from symptoms on the arms (32%) than on the face (24%), while Blacks/African Americans most often had facial symptoms (32%), followed by the arms (23%) (Supplementary Table S4).

### 3.6 Treatment

The medical curation revealed substantial treatment gaps. A significant proportion of the subjects affected by altered skin sensation, 68%, did not receive any treatment. For those who received treatment, HCPs most often prescribed antihistamines/H1 receptor blocking antihistamines (17%), followed by leukotriene receptor antagonists (LTRA; 13%), systemic corticosteroids (SCS) or hormone receptor antagonists (HRA; 11%), and local anesthetics (8%) (Figure 6).

**Figure 6:**
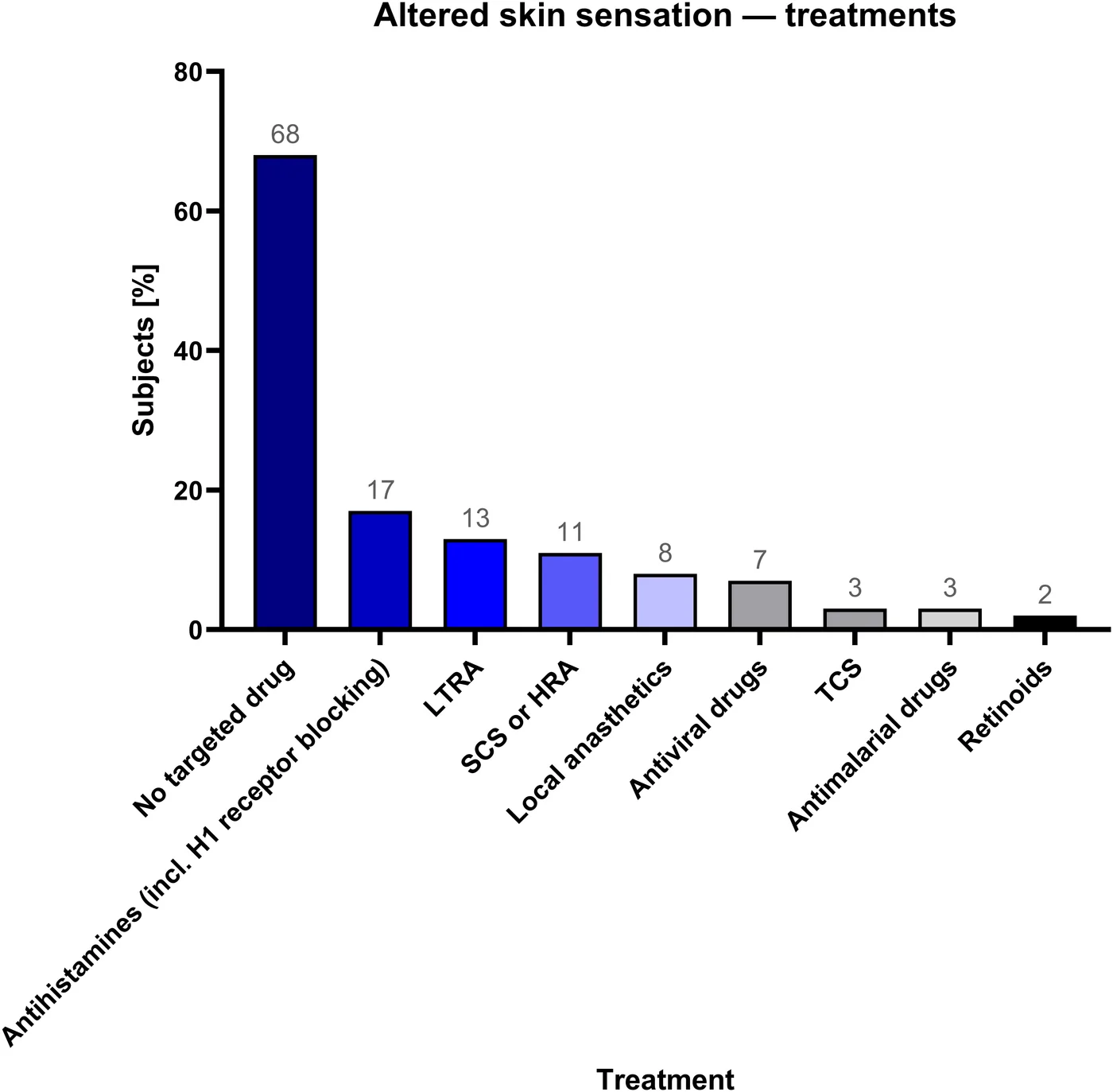
Treatment of altered skin sensation. Data from the curation of the quota-sampled subset (*N* = 240). HRA, hormone receptor antagonist; LTRA, leukotriene receptor antagonist; SCS, systemic corticosteroid; TCS, topical corticosteroid.

Patients also left the HCPs’ offices with non-prescription over the counter (OTC) recommendations (12%) and medical advice to switch to sensitive skin-specific products with no dyes/no fragrance (18%). Some were also recommended to change their diet (data not shown). Overall, the HCP's decision on a treatment option is based on their experiences and rather represents a multifaceted trial-and-error approach.

### 3.7 Psychological disturbances

It is well known that both conditions, sensitive skin and PN have a negative psychological and emotional impact. Compared to the general EHR population, the sensitive skin cohort was presented with increased comorbidities, indicating psychological distress. Six months after the index date, sleeping disorders were present in about 29% (sensitive skin cohort; Figure 1) vs. 24% (general EHR population; Supplementary Table S6), while depressive episodes occurred in 24% (sensitive skin cohort; Figure 1) vs. 20% (general EHR population; Supplementary Table S6).

### 3.8 Etiology

Based on the data from the medical curation, the underlying disease etiology of SSS was unknown in a large proportion of subjects (60%). Among those with a known cause, external contact (with products, plants, fabrics, animals, etc.) and neurological etiology were most commonly reported, followed by weather-related factors, and stress (Supplementary Table S5). The neurological diagnosis of altered skin sensation affected Hispanics most frequently and Blacks/African Americans least frequently).

## 4 Discussion

The primary goal of our RWE study was to investigate the clinical characteristics, demographics, treatment, and other factors of patients reporting sensitive skin symptoms. Categorization and interpretation of the RWD results led to the assumption that part of the patient group who reported altered skin sensation likely suffered from PN or, more specifically, from distal symmetric polyneuropathy (4) with typical symptoms such as tingling, burning, paresthesia, numbness, or from small fiber neuropathy (SFN), which is also a subtype of PN (24).

A study from 2018 showed that patients diagnosed with SSS had significantly higher neuropathic pain scores and lower heat pain thresholds than controls, suggesting that unmyelinated C-fibers may be damaged (40). One possible explanation is that the cutaneous nerve endings in patients with SSS are oversensitive or not sufficiently protected (29, 34). Likely, hyperactive transient receptor potential (TRP) channels expressed on keratinocytes play a role in SSS, which is also the case for SFN (35). Apart from the possible pathogenetic basis, SFN and SSS are both difficult to diagnose conditions, as no objective laboratory marker exists. More recent work using quantitative sensory testing has objectively characterized sensory phenotypes in skin disease, demonstrating both loss- and gain-of-function changes, including altered thermal detection, within cutaneous lesions (50). Such objective phenotyping is the kind of confirmation this hypothesis ultimately requires and is not achievable from diagnostic codes and symptom descriptors alone. Instead, patient-reported symptoms and clinical examination mainly lead to the diagnosis. Symptoms of the group herein classified as having altered skin sensation and symptoms of PN overlap, leading us to conclude that misinterpreting the symptoms has a high probability. Consistent with the gain-to-loss-of-function spectrum noted above, the paresthesia and tingling subgroup (gain-of-function) is more consistent with the small-fiber neuropathy phenotype attributed to SSS, whereas the anesthesia and numbness subgroup (loss-of-function) is more consistent with classic peripheral neuropathy. SSS is often associated with tingling or numbness, which are typical PN symptoms (21, 35). In our study, 33% of patients reported altered skin sensation, with tingling (18%) and numbness (11%) being most common. This differs from the groups categorized as having dermatitis (44%), acne (13%), or dry skin and urticaria (15%). These groups clearly suffer from skin-related conditions, most likely due to other causes, such as contact dermatitis or irritation from an external stimulus that triggers redness/erythema and/or other symptoms.

In our analysis with a special focus on the group classified with altered skin sensation, we identified several associations between the demographic characteristics of this group that support our hypothesis of misinterpretation of reported symptoms as patients may relate those symptoms to a skin condition though in reality suffer from peripheral neuropathy, potentially small fiber neuropathy. The group, which was one-third of the screened subjects with SSS across all ages, reported paresthesia (18%), anesthesia (11%), or other symptoms (4%). All these subcategories increased with age and were most prevalent in subjects aged 65 + years (paresthesia 21%; anesthesia 13%; other symptoms 5%). Considering that the prevalence of PN is known to increase strongly with age (42), this represents another commonality between the herein observed symptoms and PN and contrasts with the assumption that the prevalence of SSS tends to decrease with increasing age, at least when both sexes are considered together (32, 33).

Differences between PN and SSS supporting our hypothesis exist regarding the affected body regions. We note, however, that the pooled localization pattern, in which the face is among the most affected sites and foot involvement is low, is not consistent with a classic length-dependent peripheral neuropathy and most likely reflects the cosmetic-related and dermatological causes of facial sensitive skin. The neuropathy interpretation is therefore best restricted to the extremity-predominant subset, for which the age- and sex-stratified data (Figure 5; Supplementary Figure S2) provide support. Classically, SSS manifests particularly on the face (mostly due to the high use of cosmetics/procedures), although other parts of the body, especially the genital region, can also be affected (32, 33). PN, however, mainly affects the extremities and usually begins in a stocking-glove pattern on the feet and hands, spreading further proximally (5). Notably, we observed age-related differences in the affected body regions, with the two older (35–64 years and 65 + years) and more frequently from altered skin sensation affected groups reporting symptoms on the arms, legs, hands, and feet at least twice as often as the youngest group. Being localized in the extremities and becoming more prevalent with increasing age are typical features of PN (42).

Additionally, we found that altered skin sensation was much more common in males than in females (31% vs. 11%). Although no clear relationship with gender has been established for PN (42), individual studies identified male sex as a predictor of PN, possibly due to a higher prevalence of PN risk factors such as diabetes and obesity, as well as risk factors such as smoking (2, 43). In contrast, the prevalence of SSS appears to decline in males during aging and is higher in older females (27, 32, 33). This is also consistent with the observation that men, who were more frequently affected by altered skin sensation than women, reported symptoms in the extremities (i.e., arms, hands, legs, feet) more frequently.

In the overall SSS group, CVD (26% at index date; 50% 6 months after index date) and type 2 diabetes (12% at index date; 22% 6 months after index date) were the most common comorbidities. Since type 2 diabetes is by far the most common cause of nerve damage (4), and obesity and metabolic syndrome—both often associated with CVD—can also lead to the development of PN (44), this supports the conclusion that altered skin sensation reported in this study is related to PN rather than to a skin condition. We acknowledge, however, that a type 2 diabetes prevalence of 12% at the index date is not clearly above the expected background for a cohort in which roughly half are aged 55 or older, and therefore does not by itself indicate enrichment for diabetic neuropathy. Our interpretation is not that these are predominantly diabetic PN cases; approximately one quarter of PN is idiopathic, and the non-diabetic contributors observed here (cardiovascular and metabolic risk, smoking, and age) are also recognized causes. A modest diabetes rate is thus compatible with a mixed-etiology neuropathy. Beyond that, smoking, another risk factor for PN (45), was reported in 4% of patients at index and 14% after 6 months. Sleep disturbances (9% at index date; 29% after 6 months) are also typical comorbidities for PN patients, as symptoms worsen at night (21). Depressive episodes (7% at index date; 24% 6 months later) are also often associated with PN due to the burden of symptoms and their impact on quality of life and well-being (46); however, this is not a unique characteristic of PN, as many diseases, including dermatological conditions, are associated with psychosocial impairments such as depression, anxiety, and sleep disorders (47).

Another data point suggesting that patients may not be suffering from skin-related symptoms is the fact that the most consulted physicians were general practitioners (44%) and internal medicine physicians (9%), and only 7% were dermatologists. We hypothesize that there may have been no obvious signs of skin irritation besides the altered skin sensation, and patients may therefore have been unsure which physician to consult. In addition, 68% of patients in the altered skin sensation group did not receive any targeted treatment, suggesting the same uncertainty on the side of the physicians. Taken together, these observations raise the possibility that a subset of patients with altered skin sensation in this study had unrecognized PN that was interpreted as SSS. This is a hypothesis generated by exploratory real-world data insights and cannot be confirmed by the present analysis. This view is supported by the fact that approximately 80% of PN patients are not formally diagnosed due to several reasons, as demonstrated in recent studies with diabetic PN patients (22, 48, 49).

Alternative explanations should be considered rather than attributing altered skin sensation solely to PN. These include the localized neuropathic and compressive conditions noted in the Introduction, primary dermatological causes, psychosomatic contributors, and coding artefact arising from routine documentation. PN is one plausible interpretation among several, and the present data cannot adjudicate between them.

The reasons for the obstacles in diagnosing PN are multiple and include, on the patient side, a lack of awareness of the connection between the symptoms and their nerves, perceptions of symptoms as part of normal aging, inability to describe symptoms or—as supported by our results—misinterpretation of the symptoms. Physicians, on the other hand, may not proactively ask patients about symptoms of PN as they prioritize other complications of diabetes, such as retinopathy, nephropathy, and cardiovascular disease, due to time constraints and the lack of simple screening tools and objective measurement methods. Overall, the diagnosis of PN is often delayed by approximately five years, during which time the disease progresses and symptoms worsen, significantly impacting patients’ health and quality of life (21).

The current RWD demonstrated and confirmed existing gaps in patient care. The medical curation of patient records revealed that more than two-thirds of those affected by altered skin sensation received no treatment for these symptoms. Additionally, none of the most prescribed therapies in this group (e.g., antihistamines, LTRAs, corticosteroids) addressed the underlying symptoms of potential PN or its etiology, possibly due to misdiagnosis. Based on the curation data, approximately 14%–18% of patients continued to report relapse/worsening of their SSS over time.

There is an urgent need for early screening and precise diagnostics of PN as discussed previously (21, 23). Considering neurological symptoms in the context of skin-related complaints is a new approach that could help improve early diagnosis and treatment. Neurological signs and symptoms are often overlooked or not systematically recorded in clinical practice. This can lead to misdiagnosis, inappropriate treatment, treatment delays, and unnecessary patient burdens in different dimensions of life. Raising patient awareness of nerve-related conditions such as PN, associated symptoms, and risk factors is an important measure to improve patient care. Equally critical is educating HCPs about the importance of early diagnosis and appropriate tools that are simple yet specific, enabling them to make a diagnosis within their limited time per patient. The new data discussed here should be widely communicated among HCPs to avoid future misdiagnoses. Collaboration and closer interdisciplinary networking between specialties such as dermatology and neurology can also contribute to improved and rapid care for PN patients.

A structured medical history and targeted physical examination, supplemented by appropriate diagnostic tools, are essential for a comprehensive patient assessment. Simple screening instruments offer a promising way to detect neurological symptoms more efficiently in primary care and in the dermatological setting. As a lower-burden alternative to full sensory testing, questionnaire-based screening for neuropathic pain has been validated against quantitative sensory testing in a dermatological condition (51); the ACT (A simple sCreening Tool) is a further example of such a validated rapid instrument to identify neuropathic pain instrument (21, 23). Developing these tools and integrating them into clinical routines could facilitate early diagnosis, which is crucial for the timely initiation of individualized treatment and the prevention of disease progression, and could also help avert serious complications that burden patients, caregivers, and the healthcare system. Overall, these findings open opportunities to raise awareness through engagement with the right patient group, reduce delayed diagnosis or misdiagnosis of PN, and improve both quality of care and quality of life for those affected.

### 4.1 Strengths and limitations

RWE studies such as ours have several general strengths. First, access to data from large, diverse populations across different healthcare settings may be beneficial to enable better generalizability to broader patient populations compared to controlled study cohorts, while also allowing for sufficient inclusion of small subpopulations. Beyond that, RWE data are usually collected from routine clinical practice, which considers real-world treatment patterns (including comorbidities and comedication) and decision-making. Since RWE studies are usually retrospective and the time-consuming conduct of a prospective controlled study is not necessary, the results are usually available quickly, allowing for timely insights. However, RWE studies also have some weaknesses. Since no randomization is used, there is a considerable risk of confounding and selection bias, such as the preferred selection of complete and neglect of less complete datasets for analysis. In addition, data quality is lower than that of controlled trials as missing data, coding errors, and inconsistent recording practices are common, and certain variables, such as lifestyle factors, may not be adequately considered. The lack of standardization due to the variation in data sources, definitions, and coding systems (e.g., ICD codes) makes it difficult to compare settings across various countries or healthcare systems.

Several specific limitations should be listed. First, the analysis is exploratory: the hypothesis of an SSS to PN link was generated by inspection of these data and cannot be confirmed by the same data, and no formal test of that hypothesis was performed. Second, results derived from the quota-sampled 240-patient curation subset are descriptive and not generalizable to the full cohort. Third, retention of only complete records introduces potential selection bias. Fourth, the collection window overlapped the COVID-19 pandemic, which may have affected care-seeking, the mix of specialties consulted, and coding. Fifth, thermal sensory data, which are important for identifying small-fiber neuropathy, were not available in the source records. Sixth, the increase in comorbidity coding over follow-up could reflect either accumulating diagnostic codes or genuine new-onset disease, and the de-identified data do not permit longitudinal tracking of same individuals to distinguish the two.

Increasing awareness of this hypothesis may help improve the timely distinction of SSS from PN. Further work, including prospective and objectively phenotyped studies and continued analysis of real-world data, will be needed to test whether the association observed here is genuine and clinically actionable.

## 5 Conclusions

These new RWE data are another step toward solving the puzzle of PN diagnosis and helping multiple undiagnosed and untreated patients receive timely, targeted treatment to avoid serious and potentially life-threatening complications and maintain quality of life. The data provide new insights based on conclusive findings on how patients and physicians may misinterpret symptoms of PN, leaving up to 80% of patients undiagnosed. Understanding the patient journey and profile, along with the availability of simple screening and diagnostic tools, will enable HCPs across specialties, including dermatologists, to provide the correct diagnosis for each patient. Further studies investigating the profile of patients reporting sensitive skin will help strengthen the evidence created in our study.

## Data Availability

The results summarised in this article will be made available by the authors, without undue reservation. The raw data cannot be provided as it is not owned by the authors. Further queries can be directed to the corresponding author.
