# Supplementary material for: Beyond the skin surface: untangling sensitive skin syndrome and peripheral neuropathy through an exploratory real-world data analysis
Source: Front Pain Res (Lausanne). 2026 Oct 12;7:1883218. doi: 10.3389/fpain.2026.1883218 (PMC13696795; doi:10.3389/fpain.2026.1883218)
Supplement: Supplementary file 1 [file Datasheet1.pdf]

Supplementary Material

Beyond the Skin Surface: Untangling Sensitive Skin Syndrome and Peripheral Neuropathy through an Exploratory Real-World Data Analysis

Miranda A. Farage, Ph.D. and Dr. Inna Eiberger

Supplementary Figure S1. Study flow diagram

Attrition from the source population to the analysis cohort and the curation subset.

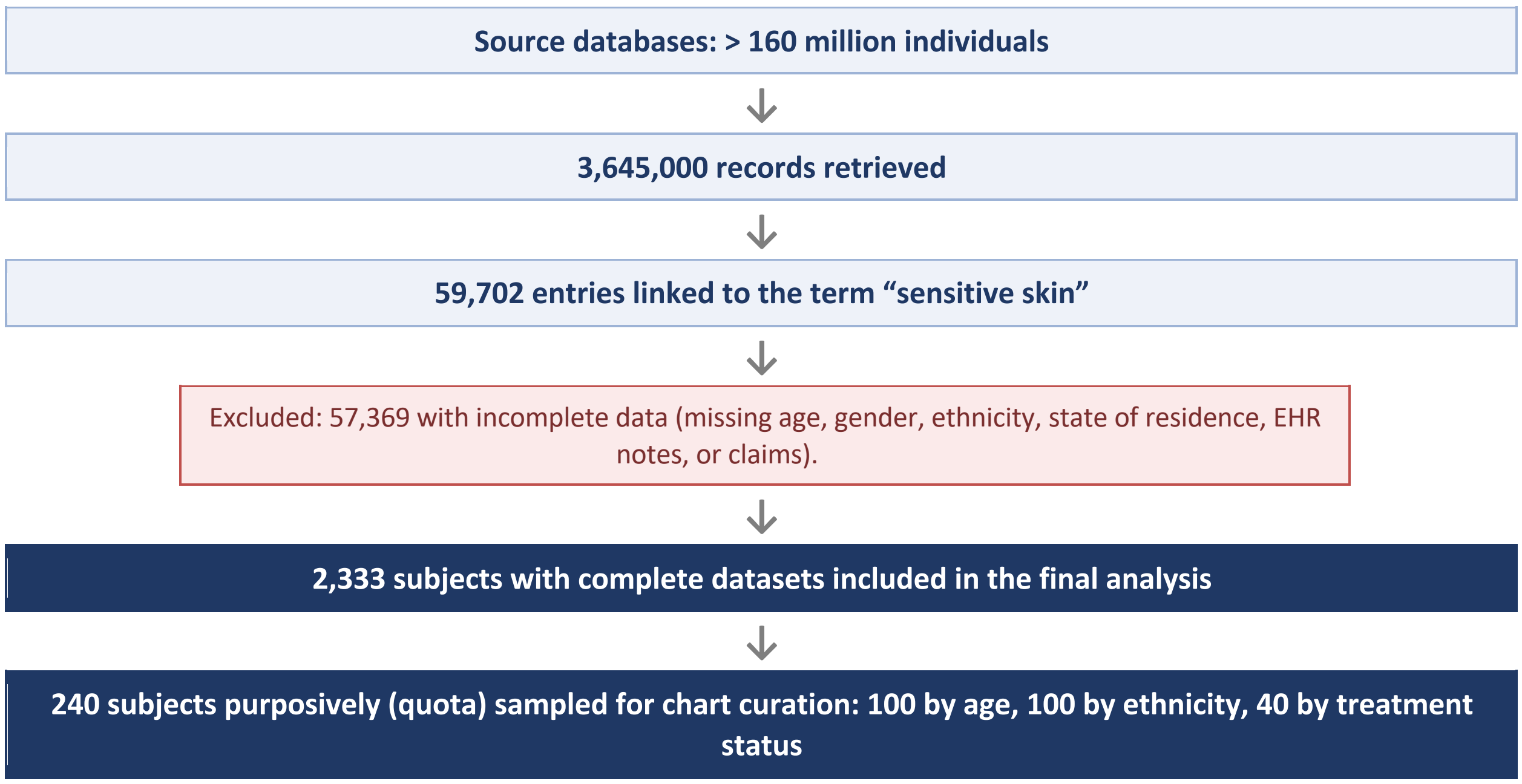

Supplementary Figure S2. Localization of symptoms by body site and age

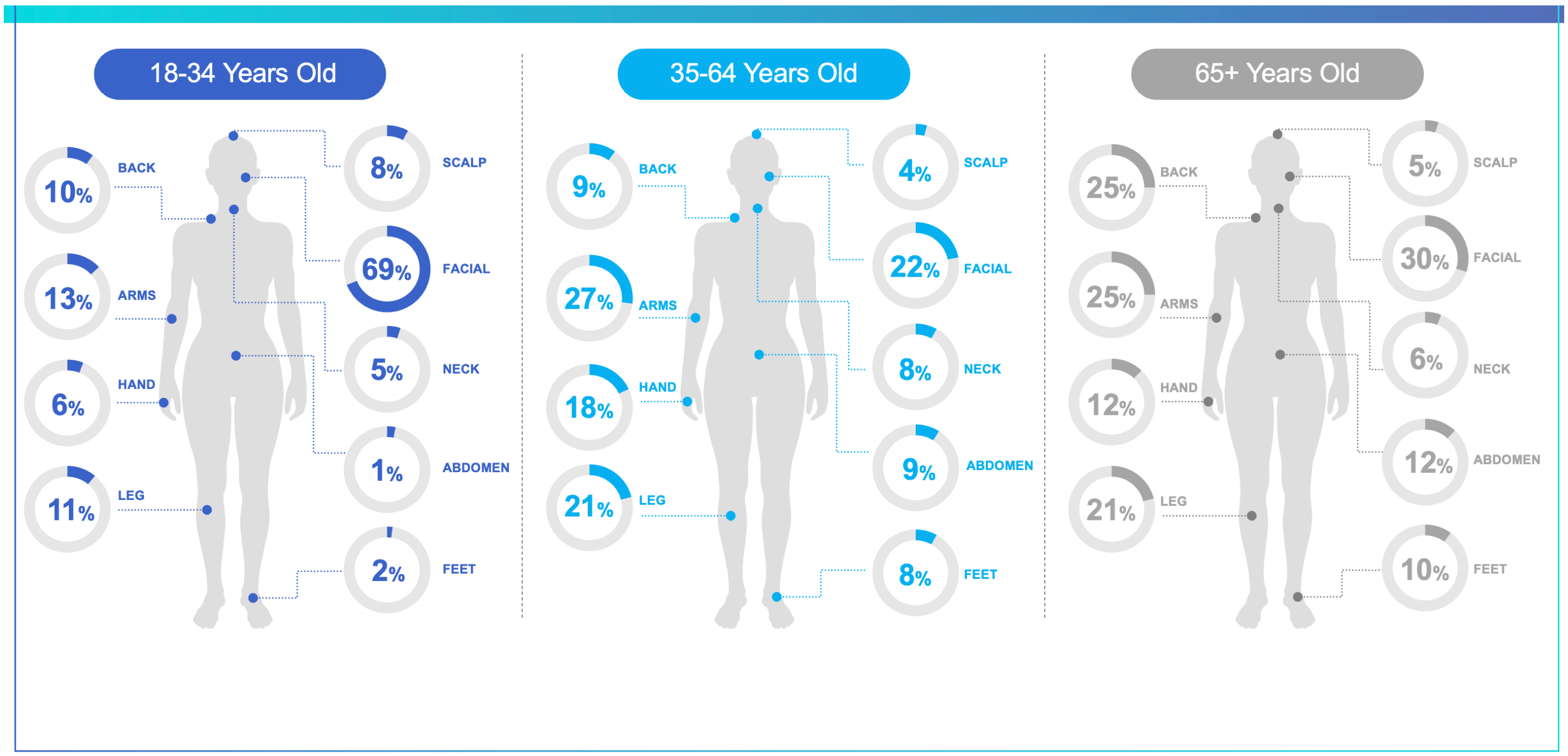

Curation subset (N=240)

Supplementary Table S3. Symptoms by ethnicity

	Itch	Rash	Redness	Pain	Altered sensation	Macules/ Papules	Raised Skin
**HISPANIC**	40%	32%	24%	20%	32%	12%	12%
**BLACK/AFRICAN AMERICAN**	35%	35%	12%	8%	4%	13%	4%
**ASIAN**	44%	32%	32%	12%	4%	20%	20%
**WHITE**	60%	40%	40%	4%	12%	28%	16%

Curation subset (N=240); values are %. Rash was reported as an even 32–40% across all groups. Note that Black/African American subjects did not rank first for any symptom.

Supplementary Table S4. Localization of symptoms by ethnicity

	Face	Arms	Legs
**Hispanic**	32	24	20
**Black / African American**	32	23	12
**Asian**	44	32	36
**White**	24	32	16

Curation subset (N=240); values are %.

Supplementary Table S5. Etiology of altered skin sensation

Etiology	n	%
**Unknown**	150	60
**External contact (products, animals, plants, fabrics, etc.)**	31	13
**Neurological**	29	12
**Medication side effects**	7	3
**Environmental / weather-related**	6	2
**Stress**	6	2
**Diet**	3	1
**Autoimmune condition**	2	1
**Metabolic (DMI or II)**	2	1
**Other**	13	5

Curation subset (N=240). Among cases with a known cause, both external contact with products and neurological etiology were the most common. The neurological diagnosis of altered skin sensation was reported most frequently in Hispanic and least frequently in Black/African American subjects. A patient might have been given more than 1 etiology.

Supplementary Table S6. Psychological comorbidities: sensitive-skin cohort vs general EHR population

Comorbidity (6 months after index)	Sensitive-skin cohort (%)	General EHR population (%)
**Sleep disturbance**	29	24
**Depressive episode**	24	20

Sensitive-skin cohort N=2,333. Values for both columns are from the manuscript; The general-population comparator is drawn from the same database used for this study.
